# Implementation of paediatric vision screening in urban and rural areas in Cluj County, Romania

**DOI:** 10.1186/s12939-021-01564-6

**Published:** 2021-12-18

**Authors:** Jan Kik, Mandy Nordmann, Simona Cainap, Mihai Mara, Daniela Rajka, Monica Ghițiu, Alin Vladescu, Frea Sloot, Anna Horwood, Maria Fronius, Cristina Vladutiu, Huibert Jan Simonsz

**Affiliations:** 1 Department of Ophthalmology, Erasmus University Medical Center, P.O. Box 2040, 3000 CA Rotterdam, the Netherlands; 2 University of Medicine and Pharmacy, Cluj-Napoca, Romania; 3 Department of Social and Medical Assistance, Cluj-Napoca, Romania; 4 School of Psychology and Clinical Language Sciences, University of Reading, Reading, UK; 5 Department of Ophthalmology, Child Vision Research Unit, Goethe University, Frankfurt am Main, Germany

**Keywords:** Vision screening, Amblyopia, Children, Implementation study, Urban, Rural, Disparity

## Abstract

**Background:**

In 2018 and 2019, paediatric vision screening was implemented in Cluj County, Romania, where universal paediatric vision screening does not yet exist. We report on the preparation and the first year of implementation.

**Methods:**

Objectives, target population and screening protocol were defined. In cities, children were screened by kindergarten nurses. In rural areas, kindergartens have no nurses and children were screened by family doctors’ nurses, initially at the doctors’ offices, later also in rural kindergartens.

CME-accredited training courses and treatment pathways were organised.

Implementation was assessed through on-site observations, interviews, questionnaires and analysis of screening results of referred children.

**Results:**

Out of 12,795 eligible four- and five-year-old children, 7,876 were screened in 2018. In the cities, kindergarten nurses screened most children without difficulties. In Cluj-Napoca 1.62x the average annual birth rate was screened and in the small cities 1.64x. In the rural areas, however, nurses of family doctors screened only 0.49x the birth rate. In 51 out of 75 rural communes, no screening took place in the first year. Of 118 rural family doctors’ nurses, 51 had followed the course and 26 screened children. They screened only 41 children per nurse, on average, as compared to 80 in the small cities and 100 in Cluj-Napoca. Screening at rural kindergartens met with limited success. These are attended by few children because of low population density, parents working abroad or children being kept at home in case of bad weather and road conditions.

**Conclusions:**

Three times fewer children were screened in rural areas as compared to urban areas. Kindergartens in rural areas are too small to employ nurses and family doctors’ nurses do not have easy access to many children and have competing healthcare priorities: there are 1.5x as many family doctors in urban areas as compared to rural areas. For nationwide scaling-up of vision screening, nurses should be enabled to screen a sufficient number of children in rural areas.

**Supplementary Information:**

The online version contains supplementary material available at 10.1186/s12939-021-01564-6.

## Background

Paediatric vision screening is generally considered an effective intervention to reduce the prevalence of persistent amblyopia among adults [1] and is common in most European countries [2]. Amblyopia is a decreased visual acuity (VA), mostly unilateral [3], caused by strabismus or refractive errors in early childhood. It can best be treated before the age of seven, with glasses, occlusion of the better eye or both [4]. Its prevalence is approximately 3.4% [5]. Unilateral amblyopia also affects the quality of life [6]. The average period of bilateral visual impairment at the end of life is much longer in children with persistent amblyopia [7].

The EUSCREEN study [8] compares paediatric vision and hearing screening programmes in Europe and aims to develop a cost-effectiveness model to assist with the introduction or modification of screening programmes, taking local circumstances into account (Additional file 1).

The European Union gives priority to “early detection by screening and follow-up for hearing, vision and speech disorders in children . . . in order to contribute to create equal educational, social and economic opportunities for children” [9].

Romania is one of a few European countries without a national paediatric vision screening programme. The country is not able to spend much on preventive healthcare, only 1.7% of health spending, as compared to an EU average of 3.1% [10]. Newborn hearing screening, for example, has high coverage in most European countries [11], but in Romania only 24% of children were born in health units where newborn hearing screening was available in 2018 [12, 15]. Vision screening has been provided by the Lion’s Club project ‘Clear view, healthy eyes’ [13] but not on a structural basis and geographically limited. Therefore, Cluj County in Romania was chosen for the implementation of a vision screening programme.

The healthcare system in Romania has been historically underfunded. Under communism, health spending was already much lower than in other Eastern bloc countries [14] and since then, though the system was transformed into a social health insurance system, health spending has remained low. In 2017, health spending in Romania was the lowest in all EU countries: 5.2% of the GDP as compared to an EU average of 9.8% [10].

Cluj County had a population of 730,216 in 2018. Cluj-Napoca is the county seat with a population of 324,276 and there are five small cities: Turda, Dej, Câmpia Turzii, Gherla and Huedin. In the county’s rural areas 251,481 people or 34% of the population resided, spread across 75 communes. In the whole of Romania, 46% of the population lived in rural areas [15].

A more detailed description of Cluj County and the Romanian healthcare system can be found in Additional file 2.

The implementation study would provide an opportunity to identify barriers and facilitators encountered when implementing a vision screening programme in a setting were no vision screening programme yet exists. A setting that is also characterised by a divide between urban and rural areas and significant healthcare disparities between these areas, thus providing an opportunity to observe whether the implementation would be affected by these disparities and, if so, in what way.

There are inequities in health and healthcare between urban and rural areas, because of a shortage of healthcare workers, lack of healthcare infrastructure, long travel distances and socioeconomic disadvantage [16]. Mindful of the World Health Organization’s position that a screening programme should “promote equity and access to screening for the entire target population” [17] and the risk that a new public health intervention may less likely be taken up by those most in need [18], this was a point of concern from the outset.

The implementation of vision screening commenced on January 1^st^, 2018 and concluded on December 31^st^, 2019. We report on the preparation of screening and the first year of implementation.

## Methods

### Implementation assessment

We used a mixed methods design to investigate the implementation, employing on-site observations, interviews, questionnaires and analysis of screening results of children. The implementation was assessed using a framework based on the work of Peters et al. [19] and Proctor et al. [20]. The implementation outcomes that were assessed and the measurement methods are displayed in Table 1.

**Table 1:** Preliminary implementation outcome variables and measurement methods (a cross denotes this method was used)

Implementation outcomes →	Acceptability	Feasibility	Appropriateness	Adoption	Fidelity	Coverage	Sustainability (will be assessed after the second year)
Measurement methods ↓							
On-site interviews with nurses	X	X	X	X	X	X	n/a
On-site interviews with family doctors	X	X	X	X			
On-site interviews with kindergarten staff	X	X	X	X			
Questionnaires for screeners	X		X		X		
Questionnaires for rural family doctors	X	X	X				
On-site observation of screening		X			X		
Data analysis of screening results				X	X	X	

Four visits were made to Romania for on-site interviews and observations. The first visit was made while preparations for screening were being made and courses for screeners took place (AH, MF), the second when screening had just started (HJS, MN), the third a few months later (HJS, MN) and the fourth after one year of screening (MF, AH, MN, JK).

Screenings were observed and screening locations throughout the county were visited, where nurses and family doctors were interviewed. When screenings were observed, the explanation and test times were measured with a stopwatch and it was observed if screening was performed according to protocol. During the fourth visit, mostly rural locations were visited, because it had become apparent that implementing screening was more difficult there. Twenty communes were visited and screeners were interviewed, as well as staff at rural kindergartens. Reports were made of the interviews, based on notes taken by multiple authors (MF, AH, MN, JK). These reports were coded and analysed.

Additionally, a questionnaire for screeners was developed, based on the questionnaire used by Tjiam et al. [21], to assess the adoption of the protocol by the screeners (Additional file 3).

From discussions with several rural family doctors it appeared that they encountered difficulties in their work that could prevent them from taking part in vision screening. To gain more insight into these difficulties, a short questionnaire was developed for family doctors (Additional file 4).

To monitor all screening results and follow-up activity, an electronic database was developed (Additional file 5). Data pertaining to all screened children were analysed to assess the results of the implementation of screening.

### Screening population

Children aged four and five are old enough for VA measurement [22] and young enough to be successfully treated for amblyopia if needed [4]. In 2018, all children born in Cluj County in 2013 and 2014 would be eligible. Altogether these were 12,795 children: 6,083 in the county seat Cluj-Napoca, 2,260 in five small cities and 4,452 in rural areas [15].

The majority of the population of Cluj County consists of Romanians (75%) but there are two substantial ethnic minority groups: Hungarians and Roma, who make up 15 and 3% according to official figures [23]. Roma are a disadvantaged group, who live in segregated communities and suffer worse health than the majority population [24]. Roma are also much less likely to have health insurance [16]. The implementation made no distinction between different ethnic groups and did not keep track of ethnicity.

A protocol for measurement of VA was developed based on the literature, the ISO 8596:2017 standard for VA measurement and expert opinion (Additional file 5). All screening examinations entered in the database were analysed to determine whether the recorded screening result was consistent with the measured VA entered in the database.

### Screening personnel and training

The programme was implemented in the city of Cluj-Napoca by the DASM (Department of Social and Medical Assistance), a municipal organisation that provides social and medical services and maintains medical offices in over 100 kindergartens and schools. In the small cities and rural areas the implementation was handled by the UMF-Cluj (University of Medicine and Pharmacy). Both set up project teams to handle their respective parts of the study. The overall local coordination of the study, as well as the development of the screening protocol and training of the screeners, was handled by the UMF-Cluj. Both the DASM and the UMF-Cluj drafted implementation plans covering objectives, target population, screening protocol, vision charts, training, care pathways and follow-up.

In Romania, the enrolment rate in rural kindergartens is 85%, as compared to 97% in urban kindergartens [25]. There are no figures on attendance, but many children attend only sporadically [26]. Because kindergartens in cities have nurses, the decision was made to train these to screen. In the public kindergartens in Cluj-Napoca, children were screened by nurses employed by the DASM. These nurses had vision screening added to their job descriptions, if they did not object.

In the small cities the nurses working at the public kindergartens were employed by the municipalities, but were contracted to screen children by the UMF-Cluj. The nurses, having a medical background, were allowed to screen children. Also, the nurses saw the children and parents every day and already had a relationship with them.

In rural kindergartens there generally are no nurses. Therefore, the implementation had to be adapted and it was decided that the rural children would be screened by the family doctor’s nurse at the family doctor’s office instead. At the time, this was considered to be the best option, also in line with the Ministry of Education and Ministry of Health’s joint regulation 5298/2011: “Where there are no medical and dental practices in kindergartens and schools, the medical assistance . . . shall be performed by doctors and dentists from the respective localities or from nearby localities.” The rural family doctors’ nurses were also contracted by the UMF-Cluj.

All nurses who screened children received €7,- gross (around €4,- net) per child screened in addition to their salary.

A training programme for nurses and doctors was organised by the UMF-Cluj with support from two of the authors (AH, MF). Three courses were organised in the city of Cluj-Napoca in late 2017 with each course consisting of six hours both on Saturday and on Sunday. Because participation of nurses from the rural areas was low, the original implementation plan was adapted and two additional courses were organised in 2018. Travel expenses to Cluj-Napoca were not reimbursed though, nor was lodging for the two-day course.

Development of the course curriculum was supervised by one of the authors (CV) and was credited with twelve points of Continuing Medical Education. The course included theory on vision problems in children as well as practical instructions on how to measure VA. Participants had to pass a test to be allowed to screen children.

Because in the first six months of the implementation very few children had been screened in the rural areas, the protocol was modified by adding an alternative method of screening in August 2018: the family doctors’ nurses were advised to visit the rural kindergartens to screen the children there.

### Information for parents and children

Parents’ awareness of the importance of early detection of amblyopia and their attitude towards screening, are relevant to the success of a screening programme [27]. To inform parents about the purpose of screening, leaflets and posters were developed that explained the study in lay terminology. To explain the screening procedure to the children, a cartoon was developed.

In adherence to the Declaration of Helsinki [28], parents’ informed consent was obtained before screening. Parents signed a consent form, approved by the UMF-Cluj’s Ethics Committee, that included detailed information about the purpose, objectives and procedures of the study and made it clear they could opt in or opt out.

After screening, the result was explained to the parents by the screener. When the child needed to be tested again, a re-examination was scheduled. In case of referral, the screeners were to instruct the parents to go to their family doctor for referral to an ophthalmologist. They were also to give the parents a list of ophthalmologists (specified below), a form with a summary of the screening result and a form for the ophthalmologist to report back the results of the examination. As it was not possible to grant the ophthalmologists access to the project database, the parents had to return the filled-out form to the screener or to the kindergarten staff, who then had to return the filled-out form to the DASM or the UMF where secretaries entered the forms in the database.

### Referral

Once low visual acuity had been detected, amblyopia, or another ophthalmological condition, needed to be diagnosed and treated. All ophthalmologists in Cluj County were sent a letter by the UMF-Cluj about the implementation of vision screening. They were asked whether they were willing to examine and treat children who had screened positively. Ophthalmologists who responded affirmatively, were included in the list given to parents when their child was referred. Diagnostic assessment by orthoptists was not an option because there are no orthoptists in Romania.

While the Romanian health insurance covers a visit to an ophthalmologist, it does not cover occlusion patches or glasses [29] and treatment was not funded by the study. On average, amblyopia treatment requires two pairs of glasses and around 500 patches, roughly estimated. Glasses cost, depending on specifications, about RON 500,- (€103,-) on average, but are available from around RON 140,- (€29,-). A patch costs about RON 2.45 (€0.50). The price of glasses and patches could be high for low-income parents, considering the average monthly net salary in Cluj County in 2018 was RON 3,026,- (€621,-) even though this was 15% above the Romanian average [15].

## Results

### Screening personnel and training

**Fig. 1:**
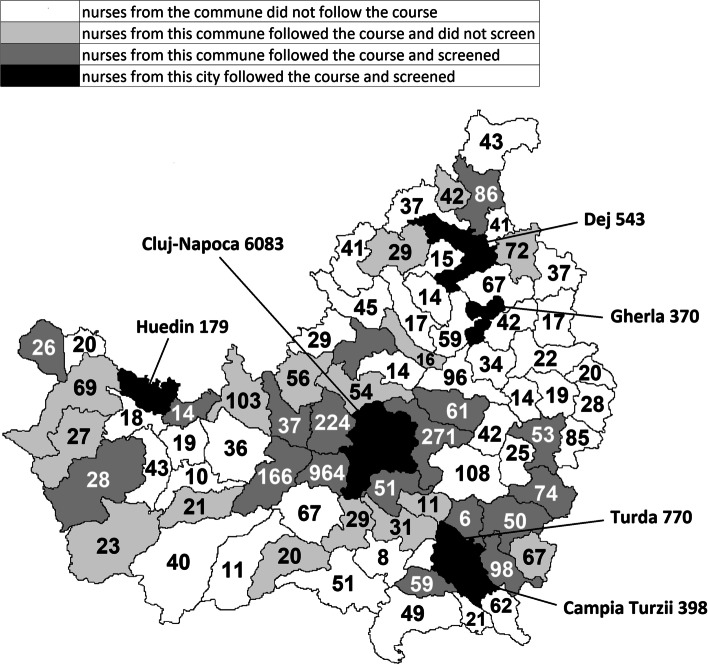
County Cluj map with eligible children per commune or city in 2018. Note that in 41 out of the 75 rural communes, none of the family doctors' nurses had followed the vision screening course

Three courses for screeners were held in late 2017. Two additional courses were organised in 2018 because participation in the initial courses from rural areas had been low. Altogether, the courses were attended by 233 persons (154 nurses and 79 doctors). All passed the concluding certified test, but only 97 (42%) screened children. This was for a large part because all but one of the doctors did not screen children (Fig. 1). It was considered useful for doctors to attend the course, even when nurses were to screen the children, so they could supervise the screening.

**Fig. 2:**
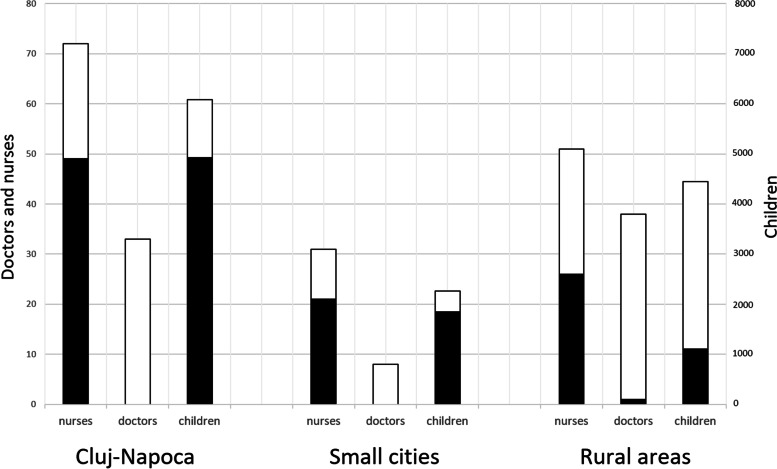
Nurses and doctors who followed the course (left) and eligible children (right) in Cluj-Napoca, small cities and rural areas. Black are the proportions that screened or were screened, respectively

The majority of nurses from the cities who followed the course, went on to screen children. From the city of Cluj-Napoca, 72 nurses attended the course, 49 (68%) of whom screened children (Fig. 2). From the small cities, 31 nurses followed the course, 21 (68%) of whom screened children. From the rural areas nurses from 34 out of 75 communes (45%) followed the course. Nurses from 18 communes (24%) went on to screen children. Nurses who followed the course were from communes with larger numbers of eligible children than nurses who did not follow the course (on average 87, as compared to 37).

From a total of 118 rural family doctors’ nurses, 51 (43%) followed the course and 26 (22%) screened children. The fact that less than half the rural nurses, representing less than half the rural communes, followed the course, put screening in rural areas at a disadvantage before screening even started.

The average number of children screened by one person in the rural areas was 41, as compared to 80 in the small cities and 100 in the city of Cluj-Napoca. This meant that nurses in cities could build up their screening expertise more quickly, as compared to nurses in rural areas.

Analysis of the screening data after six months of screening showed that coverage in rural areas was very low in comparison to the cities: children had been screened in only 15 out of 75 communes and only 0.28x the average birth rate had been screened in the rural areas, as compared to 1.19x in Cluj-Napoca and 1.43x in the small cities.

According to the interviews with the family doctors’ nurses, the main problem with screening at the doctors’ offices was that parents would not bring their children for screening, even when invited repeatedly. Therefore, in August 2018 the nurses were advised to screen children at the rural kindergartens as an alternative. Additionally, they were encouraged to visit kindergartens in neighbouring communes to screen children, if no screening would take place there otherwise. Eventually five nurses screened children in more than one commune.

A schematic overview of all the assessed implementation outcomes, in relation to the various measurement methods, is presented in Table 2.

**Table 2:** preliminary assessment of the implementation outcomes after one year.

**Acceptability**		
On-site interviews with nurses, family doctors and kindergarten staff	Urban & rural	Vision screening was considered as important by the majority of nurses, family doctors and kindergarten staff.
Questionnaires for screeners	Urban & rural	The majority of nurses expressed a positive attitude towards screening and indicated they believe screening is important and should be provided to all children.
Questionnaires for family doctors	Rural	All rural family doctors considered vision screening important.
**Feasibility**		
On-site interviews with nurses	Urban	The kindergartens were a practical setting, because the nurses see a lot of children every day and know the children and their parents well.
	Rural	The family doctors’ offices were not a practical setting, because parents did not bring their children. Some nurses did not have the time to screen. Screening in rural kindergartens was hampered by low attendance and travel distances.
On-site interviews with family doctors	Rural	Most family doctors indicated their nurses lacked time to screen and do the paperwork involved.
On-site interviews with kindergarten staff	Urban	Kindergarten nurses could screen large numbers of children.
	Rural	Kindergartens were considered less practical because of a lack of nurses and the low numbers of children attending.
Questionnaires for family doctors	Rural	Lack of funds, too many patients, personnel costs, travel time to patients in remote areas would make screening difficult.
On-site observation of screening	Urban	Nurses were able to carry out screening according to protocol in the kindergartens.
	Rural	Nurses were able to carry out screening according to protocol in family doctors’ offices and kindergartens. On few occasions, available spaces were too small to measure visual acuity.
**Appropriateness**		
On-site interviews with nurses	Urban	Most parents reacted well to the idea of vision screening.
	Rural	Some parents reacted well, but others were not interested in vision screening at all.
On-site interviews with family doctors	Rural	Most family doctors felt they had too much other preventive healthcare priorities and that parents lack awareness of the benefits of preventive healthcare.
On-site interviews with kindergarten staff	Urban	Screening by nurses in kindergartens was considered suitable for the setting. Most parents reacted well to the idea of vision screening.
	Rural	Opinions among kindergarten staff were divided. Some said parents were positive about vision screening while others said parents were negative.
Questionnaires for rural family doctors	Rural	Some family doctors said parents would be positive about vision screening, others said they would not.
**Adoption**		
On-site interviews with nurses	Urban	Introducing vision screening was considered a good idea and were enthusiastic to participate.
	Rural	Some nurses were enthusiastic to participate in screening, but others said they lacked the time to do so.
On-site interviews with family doctors	Rural	Most family doctors indicated their nurses did not have to time to take up screening and do the paperwork involved.
On-site interviews with kindergarten staff	Urban & rural	Vision screening was considered a good idea and staff were inclined to cooperate.
Questionnaires for screeners	Urban & rural	The majority of nurses considered vision screening a natural part of their work.
Data analysis of screening results	Urban	More than two-thirds of the nurses who followed the course participated in vision screening.
	Rural	Only 22% of all nurses participated in vision screening.
**Fidelity**		
On-site interviews with nurses	Urban & rural	Nurses said they were able to perform screening adequately, though some mentioned it was a bit difficult in the beginning.
Questionnaires for screeners	Urban & rural	Nurses felt confident they were able to screen.
On-site observation of screening	Urban & rural	Most nurses performed screening according to protocol.
Data analysis of screening results	Urban & rural	There were many outliers among nurses when it came to referral rates – both very low and very high referral rates – and also other indications that the protocol was not always followed correctly.
**Coverage**		
On-site interviews with nurses	Urban	Most parents consented to having their child screened.
	Rural	Most parents did not bring their children to the doctor’s office for screening. Most family doctors’ nurses who went on to screen in kindergartens said that they only found small numbers of children there.
Data analysis of screening results	Urban	81% (Cluj-Napoca) and 82% (small cities) of eligible children were screened.
	Rural	25% of eligible children were screened. Screening took place in 24 out of 75 rural communes.

### On-site interviews

In January 2019, 34 professionals were interviewed: thirteen family doctors, fourteen nurses (ten of whom screened children, four of whom did not) and seven kindergarten staff. The urban kindergarten nurses indicated that screening was not difficult because they saw the children on a daily basis, meaning there were no difficulties in scheduling screening. Also, the urban kindergartens are attended by many children, so many children could be screened in a short time.

According to both urban and rural screeners, most parents consented. Only a few refused, reportedly out of fear screening might hurt their child, because the child had already been diagnosed with an eye condition, or without providing a reason.

The rural family doctors’ nurses said that it was very difficult to get parents to bring children to the doctor’s office for vision screening. Parents often had other priorities, did not understand the benefits of preventive healthcare, did not think there was anything wrong with their children’s eyes or did not seem interested. There were also several nurses and doctors who mentioned they lacked time to screen and do the paperwork involved.

The family doctors’ nurses who screened at the rural kindergartens, said this worked better, but was not without problems either. The number of children attending the rural kindergartens is usually lower than the number enrolled there. According to kindergarten staff, this is because many parents who go abroad to work, take their children with them or leave their children with grandparents who often live in a different commune. Also, many rural children go to kindergartens in cities, because their parents work there. In winter, there are even fewer children, because many stay at home due to seasonal illnesses, weather or road conditions.

Some nurses and doctors seemed unconvinced or not aware of the benefits of vision screening or did not want anything to do with it for reasons that were unclear. Others were very interested in screening. In the interviews, nurses and doctors who were more knowledgeable about amblyopia also expressed more willingness to participate in vision screening.

Several interviewees who work in communities with a substantial Hungarian population mentioned a specific problem in reaching this group was language. An issue encountered in communities with a substantial Roma population was a greater lack of awareness of the benefits of preventive healthcare than among the general population.

Views among nurses and doctors differed as to whether parents would take referred children to an ophthalmologist and, if necessary, buy patches or glasses. Most said this would depend on parents’ affluence, though awareness of its necessity was also mentioned as a relevant factor. There was little experience with follow-up in rural areas in the first year of screening though, because few children were screened (see Additional file 6 for a more detailed report on the on-site interviews).

### On-site observations

Screenings of fourteen children at seven locations were observed by one of the authors (MN) during the first weeks of implementation. Most steps were carried out in accordance with the screening protocol. All screenings were performed in a separate room, where a three-metre distance to the VA chart was marked on the floor with tape. All screenings were performed by nurses, either at a doctor’s office or a kindergarten, mostly using the Tumbling E chart. Six children were four years old, eight children were five years old.

Explaining the test took 1:20 minutes (SD = 48 seconds), on average. In twelve cases, the test was explained before the measurement started. In the other two cases the test was explained while the child was already wearing the spectacle frames with unilateral cover, but before showing the chart. In thirteen out of fourteen screenings the child indicated the position of the optotype by pointing its direction. In one case the child explained the direction with words.

The average total time for screening, including explanation, was 8:20 minutes (SD = 2 minutes). Seven out of fourteen children were referred, in four cases because of a VA difference between the eyes or low VA in one eye and in three cases because of low VA in both eyes.

### Questionnaires for screening nurses

Eight questionnaires for screeners were completed, out of 25 distributed. The respondents expressed a positive attitude towards screening and indicated they believe screening is important and should be provided to all children. They felt confident and able to participate in the programme.

After twelve months the questionnaire for screeners was distributed among 40 screeners and this time 23 were returned. The attitude of the screeners was similar to the one expressed in the previous questionnaire. They again indicated vision screening is important and should be provided to all children and they considered screening an evident part of their work.

### Questionnaires for family doctors

The questionnaire for rural family doctors was sent to 98 doctors and completed by 23 respondents. They were very positive about vision screening and most considered screening for children aged four and five to be very important. However, all respondents mentioned many different problems affecting their own work. Most often mentioned, 14 times, was the lack of reform of the national healthcare system. Various other, mostly cost-related problems were also mentioned several times: low payments by insurance, too few diagnostic tests reimbursed and personnel costs. Also mentioned were workload, a lack of awareness of the importance of vision screening among medical professionals as well as among the general population and too many administrative tasks (more details can be found in Additional file 6).

### Screening coverage

**Fig. 3:**
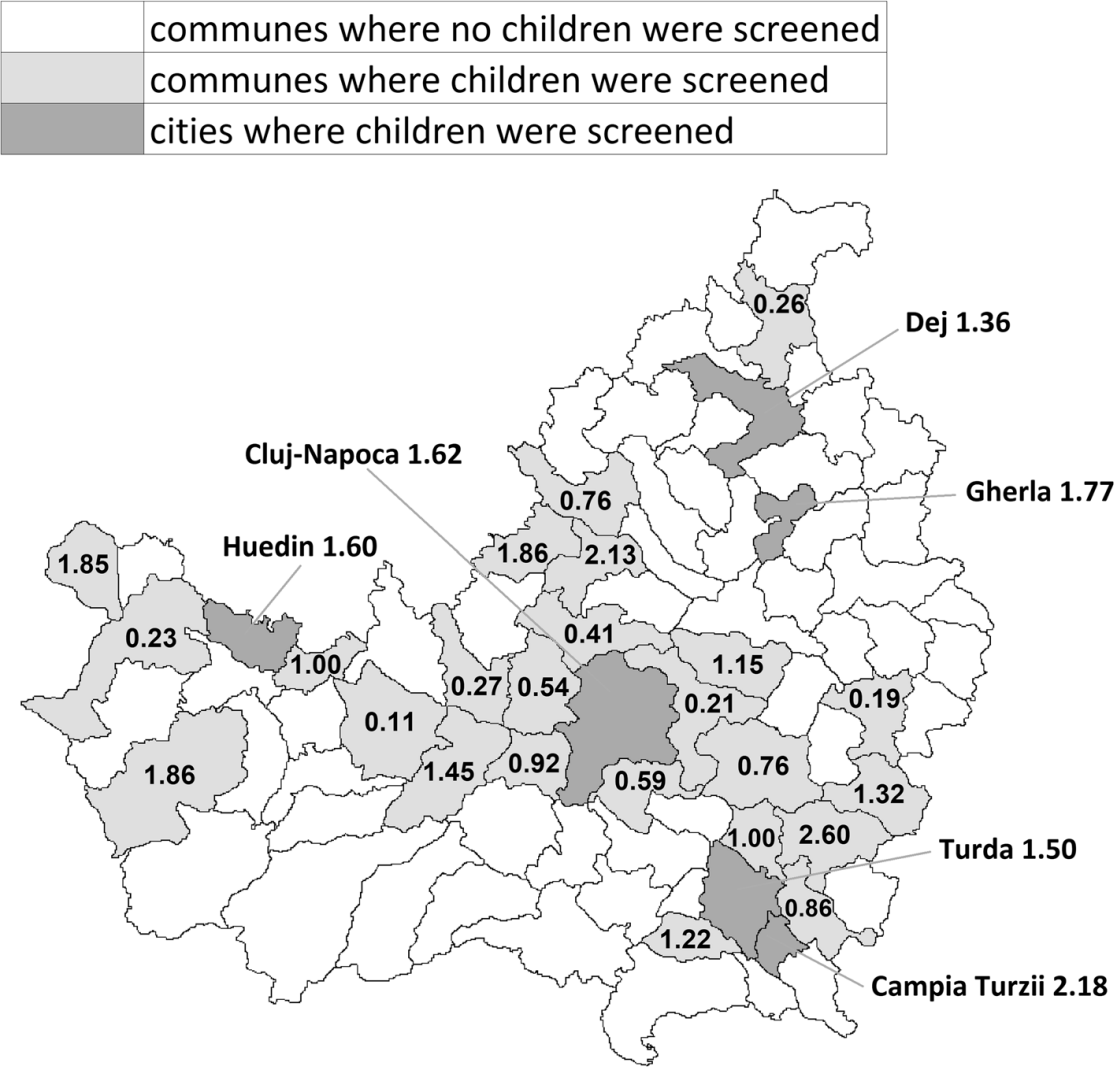
Number of four- and five-year-old children screened in 2018, divided by the average birth rate in each city or rural commune

In 2018, 7,876 children were screened or 1.23x the average birth rate (two birth years being eligible). In the city of Cluj-Napoca 4,928 children were screened, in the small cities 1,848 and in the rural areas 1,100. This means that in Cluj-Napoca 1.62x the average birth rate was screened, in the small cities 1.64x and in the rural areas 0.49x. Children were screened in 24 out of 75 rural communes, that together comprised 54% of the rural population. Even so, in the 24 communes where children were screened, only 0.84x the birth rate was screened. An overview of how many children were screened is presented in Fig. 3.

It should be noted that children were not always screened in the locations where they were residents, as it is not uncommon for rural children to attend a kindergarten in a city. An investigation brought to light that 589 children from rural areas were screened in cities: 436 in Cluj-Napoca and 153 in small cities. If the figures are adjusted to take this into account, from Cluj-Napoca 1.48x the birth rate was screened, from the small cities 1.50x and from the rural areas 0.76x.

### Referral to family doctor and ophthalmologist

The overall referral rate was 14%. It was highest in Cluj-Napoca, 15% as compared to 11% in the small cities and 10% in the rural areas. There were large differences between screeners, with referral rates varying from 0 to 44%. One exceptional screener had a referral rate of 76%. This was explained by the fact this screener screened children from a minority group, many of whom, according to the screener, did not pass because they did not understand the test.

Referral decreased as screeners gained experience, from 20% in January to 12% in March. The number of children scheduled for a repeat screen was only 2.4%.

Out of 97 screeners, 28 had a referral rate above 15% and 32 had a referral rate below 5%. Of these, fifteen did not refer a single child, even though they screened 45 children on average. Three of these screened more than 92 children. Not referring a single child out of more than 92 screened is highly implausible: assuming a prevalence of amblyopia of 3.2%, the chance of a nurse not referring any children is less than 5% as soon as the number of examined children exceeds 92, according to the formula (HP Prime) BINOMIAL_CDF (93, 0.032, 0) = 0.0486, which is slightly less than alpha = 0.05. One other nurse, who screened 86 children, recorded exactly the same visual acuity in both eyes for all these 86 children screened in 2018.

### Outcome of screening

The outcome registered by the screener (pass, repeat or refer) was checked against the measured VA. Out of 7,876 screenings, in 61 cases (0.8%) VA was recorded as below the threshold, and the screener nevertheless recorded a pass. Conversely, in 12 cases (0.2%) where the recorded VA did not warrant referral, the result was recorded as a referral nevertheless. It should be noted that we do not know whether these children were indeed erroneously either referred or not referred, or whether the outcome was entered incorrectly in the database.

**Fig. 4:**
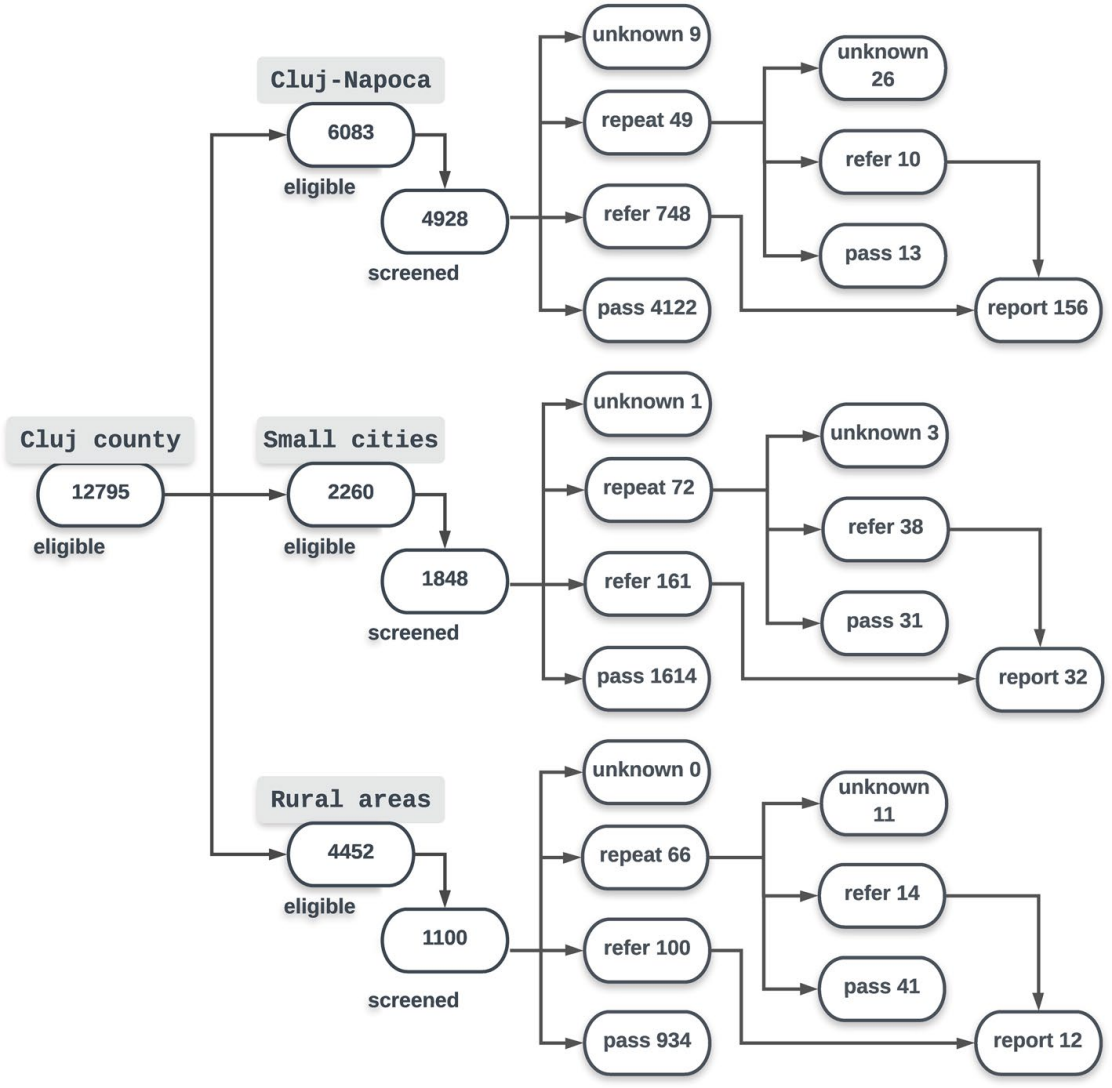
screening results in 2018. Eligible children were children born in 2013 and 2014.

Out of 187 (2.4%) repeat screens, 46% of children passed the repeat screen while 33% were referred to an ophthalmologist. In 21% of cases a repeat screen was not performed or the result was not entered in the database. Out of 1,071 children referred altogether, in 200 (19%) cases a report from the ophthalmologist was entered in the database: for 21% of children referred in Cluj-Napoca, 16% in the small cities and 11% in rural areas. On average, these 200 children were examined 32 days after they were screened. The flow chart of referrals is presented in Fig. 4.

There may be several reasons why in relatively few cases a diagnostic report was entered in the database. A substantial number of parents may not have taken their child to an ophthalmologist in spite of the child being referred. In the rural areas there are no ophthalmologists, meaning parents from rural areas had to take their child to a city for an ophthalmological assessment and repeat visits for treatment of amblyopia. Almost twice as many ophthalmological reports from Cluj-Napoca as from the rural areas were entered in the database: 21% as compared to 11%. From the small cities this was 16%. However, a substantial number of children may have seen an ophthalmologist without the results of their examinations having been reported back.

## Discussion

This study investigated the first year of the implementation of paediatric vision screening in Cluj County. Screening was implemented successfully in urban areas, where children were screened by nurses in kindergartens. In rural areas, where there are no nurses in kindergartens, the alternative chosen, screening by family doctors’ nurses at the family doctors’ offices, did not work well.

The urban kindergartens were attended by many children, meaning the nurses could screen a large number of children in a short time and quickly develop screening proficiency. Children were screened by nurses they see every day and know and trust. The nurses also saw the parents on a daily basis, making it relatively easy to explain screening to parents and to hand out and collect consent forms.

In the rural areas, there are no nurses in the kindergartens because the kindergartens are attended by few children. Rural kindergartens in Romania also have difficulties recruiting staff [24]. In Cluj County, there was one kindergarten teacher per 402 inhabitants in urban areas as compared to 723 in rural areas in 2018 [15]. Therefore, children were to be screened by the family doctors’ nurses at the family doctors. However, less than half the family doctors’ nurses followed the screening course, representing less than half the rural communes. Participation may have been low because, though the course itself was free, nurses had to travel to Cluj-Napoca for it and neither travel expenses nor lodging were reimbursed, whereas the courses were held on Saturday and Sunday.

Contrary to their urban counterparts, almost half of the rural nurses who attended the course, did not screen children. Some did not want to screen, even though they had followed the course, because they were too busy to do the paperwork.

The implementation of vision screening in rural areas is hampered by existing inequities in healthcare: a lack of healthcare infrastructure, competing preventive healthcare priorities, and lack of awareness among parents. Crucial is a lack of access to many children at the same place at the same time because of long travelling distances, low kindergarten attendance because of low population density, parents working abroad or children staying home because of illness or weather or road conditions.

Health services in rural areas are unable to cope with demands [30]. There is a shortage of doctors in Romania that is far worse in rural than in urban areas. In Cluj County there is one family doctor per 1,559 inhabitants in urban areas as compared to one family doctor per 2,372 inhabitants in rural areas (a disparity ratio of 1.52). Two rural communes in Cluj County have no family doctor at all [15]. For one-third of the rural population, reaching a doctor’s office requires more than 30 minutes of travel one-way [31]. The mortality rate in Cluj County in 2018 was 10.4 in urban areas as compared to 13.9 in rural areas and life expectancy 78.5 as compared to 75.6 [15]. In the whole of Romania, health insurance coverage was 94.9% in urban areas as compared to 75.8% in rural areas in 2014 [16]. Vision screening also has to compete for limited resources with other forms of preventive care, such as vaccinations and hearing and development screening.

Importantly, it was difficult the reach the eligible children and especially to find larger groups of children together, which was much easier in the cities where the kindergartens are attended by many children. In rural areas nurses screened only 41 children per nurse, on average, as compared to 80 in the small cities and 100 in Cluj-Napoca.

Participation of parents was hampered by a lack of awareness of the benefits of preventive healthcare in general. According to the rural nurses, understanding of preventive healthcare is low and most people in the rural areas only go to the doctor when they are ill. A quarter of Cluj’s rural population has a limited level of health literacy [32]. Nurses also mentioned a lack of knowledge of amblyopia among the rural population. The issues encountered in the rural areas are discussed in greater detail in Additional file 7.

The evaluation of a cervical cancer screening pilot in Cluj County found similar disparities between rural and urban areas: participation among family doctors in rural areas, where there are no gynaecologists, was very low and it was difficult to reach women in isolated areas [33]. A study of a national cervical cancer screening programme in Romania found that the penetration of the programme in rural areas was almost non-existent [34]. Women in urban areas in Romania also had more knowledge of cervical cancer prevention as compared to women from rural areas and were more than three times as likely to get screened for cervical cancer [35]. The aforementioned study found that employing a mobile screening unit was an efficient solution to the issues encountered in rural areas [33].

Inequitable access to screening is a common issue in preventive healthcare [36] and a consistent finding across various screening programmes is that participation is lowest among the socially most deprived [37]. However, financial barriers are a less important impediment to screening than education, having health insurance and having a regular physician [38].

An implementation study of vision screening in Peru [39] encountered similar issues in remote rural areas, but found that it was possible to deliver a more equitable programme by offering additional support to poor, remote communities, taking into account local geographical and socioeconomic needs. This did require additional measures were taken that had not been included in the original implementation plan, such as a mobile unit to reach children in remote locations and financial support for parents who could not afford transport, treatment or glasses.

## Conclusions

Paediatric vision screening by resident nurses in kindergartens was implemented successfully in urban areas in Cluj County, but in rural areas, screening by family doctors' nurses  was less successful. In the cities, more than three times as many children were screened as compared to rural areas and In 51 out of 75 rural communes, no screening took place in the first year.

Screening in rural areas was hampered by a lack of healthcare infrastructure and personnel, competing preventive healthcare priorities and lack of easy access to many children because of long travel distances and low kindergarten attendance because of low population density, parents working abroad or children staying home because of illness or weather or road conditions.

These findings are consistent with existing health and healthcare disparities between rural and urban areas concerning insurance coverage and developmental and educational opportunities. For future nationwide scaling-up, the urban kindergarten model is suitable but in the rural areas screeners should be enabled to screen a sufficient number of children.

## Supplementary Information

**Additional file 1.** Cost-effectiveness model. Description of the cost-effectiveness model.

**Additional file 2.** Cluj County and Romanian healthcare. Short characterisation of Cluj County and the Romanian healthcare system.

**Additional file 3.** Questionnaire for screeners. The questionnaire that was distributed among screeners.

**Additional file 4.** Questionnaire for rural family doctors. The questionnaire that was distributed among rural family doctors.

**Additional file 5.** Protocol for measurement of visual acuity, screening protocol and database. A description of the protocols used and the database used to record screening data.

**Additional file 6.** On-site interviews and questionnaires. Extended analysis of the interviews conducted on-site and the questionnaires distributed among screeners and rural family doctors.

**Additional file 7.** Barriers in rural areas. Extended discussion of the barriers encountered in rural areas.

## Data Availability

The datasets generated and analysed during the current study are not publicly available due to privacy considerations.
