# Supplementary material for: Implementation of paediatric vision screening in urban and rural areas in Cluj County, Romania
Source: Int J Equity Health. 2021 Dec 18;20:256. doi: 10.1186/s12939-021-01564-6 (PMC8684067; doi:10.1186/s12939-021-01564-6)
Supplement: Supplementary file 1 — Additional file 1. Cost-effectiveness model. Description of the cost-effectiveness model. [file 12939_2021_1564_MOESM1_ESM.docx]

**Additional file 1: cost-effectiveness model**

The EUSCREEN study [1] compares paediatric vision and hearing screening programmes in Europe and aims to develop a cost-effectiveness model to assist with introduction, modification or disinvestment of screening programmes, taking local circumstances into account. Alongside the development of the model and informed by its preliminary predictions, a vision screening programme was implemented in the county of Cluj in Romania. The aim was for the model, taking all relevant local circumstances into account, to calculate the most optimal screening programme for any country. In the short preparation time for the implementation in Cluj County, however, it proved not possible for the model to calculate the most optimal screening programme.

A micro-simulation model was developed using the MIcrosimulation SCreening ANalysis (MISCAN) model structure [2]. This model, developed to simulate the natural history of diseases and to evaluate screening programmes, was adapted to evaluate vision screening programmes. In the preparation phase in 2017, the model was used to evaluate several possible vision screening programmes, taking into account local circumstances in Cluj County such as demography and geography, natural history of vision impairment in Romania and costs. It had been our original intention to have the model predict the most cost-effective screening protocol for Cluj County. However, the model was not sufficiently developed when the decision on the protocol had to be made and the training of screeners began. Furthermore, because of sparse data on demographics, current provision of health care, follow-up and costs, it proved difficult to adapt the model specifically for the situation in Cluj County and it was not possible for the model to take all of the local circumstances into account to calculate the most cost-effective vision screening programme. As a consequence, decisions made for the screening programme relied much on sound clinical judgement and experience in implementation of vision screening in other countries.

**References**

1. Euscreen. <https://www.euscreen.org>. Accessed 3 September 2020.

2. Habbema JD, Oortmarssen GJ van, Lubbe JT, Maas PJ van der. The MISCAN simulation program for the evaluation of screening for disease. Comput Methods Programs Biomed. 1985;20(1):79-93.
