# Supplementary material for: Implementation of paediatric vision screening in urban and rural areas in Cluj County, Romania
Source: Int J Equity Health. 2021 Dec 18;20:256. doi: 10.1186/s12939-021-01564-6 (PMC8684067; doi:10.1186/s12939-021-01564-6)
Supplement: Supplementary file 2 — Additional file 2. Cluj County and Romanian healthcare. Short characterisation of Cluj County and the Romanian healthcare system. [file 12939_2021_1564_MOESM2_ESM.docx]

**Additional file 2: Cluj County and Romanian healthcare**

**Cluj County**

The county of Cluj in north-western Romania had a population of 730,216 in 2018 [1]. The city of Cluj-Napoca is the county seat with a population of 324,276. There are five small cities: the municipalities of Turda (population 55,907), Dej (38,250), Câmpia Turzii (27,745), Gherla (23,002) and the town of Huedin (9,564). In the county’s rural areas 251,481 people or 34% of the population reside, spread across 75 communes (groups of villages). In the whole of Romania, 46% of the population lives in rural areas (Supplementary figure 1).

There are many differences between the rural communes: some communes are rural in name but are, in reality, more like suburbs. Florești for example, neighbouring Cluj-Napoca, has more inhabitants than most of the small cities. Other communes are indeed located remotely and inhabited by small populations spread over a relatively large area.

Population density in the communes varies from 6 per square kilometre (Belis, Valea Ierii) to 599 (Floresti). The average population density in the 75 communes is 49 per square kilometre.

Supplementary figure 1: map of Romania indicating the percentage of the population living in rural areas in every county.


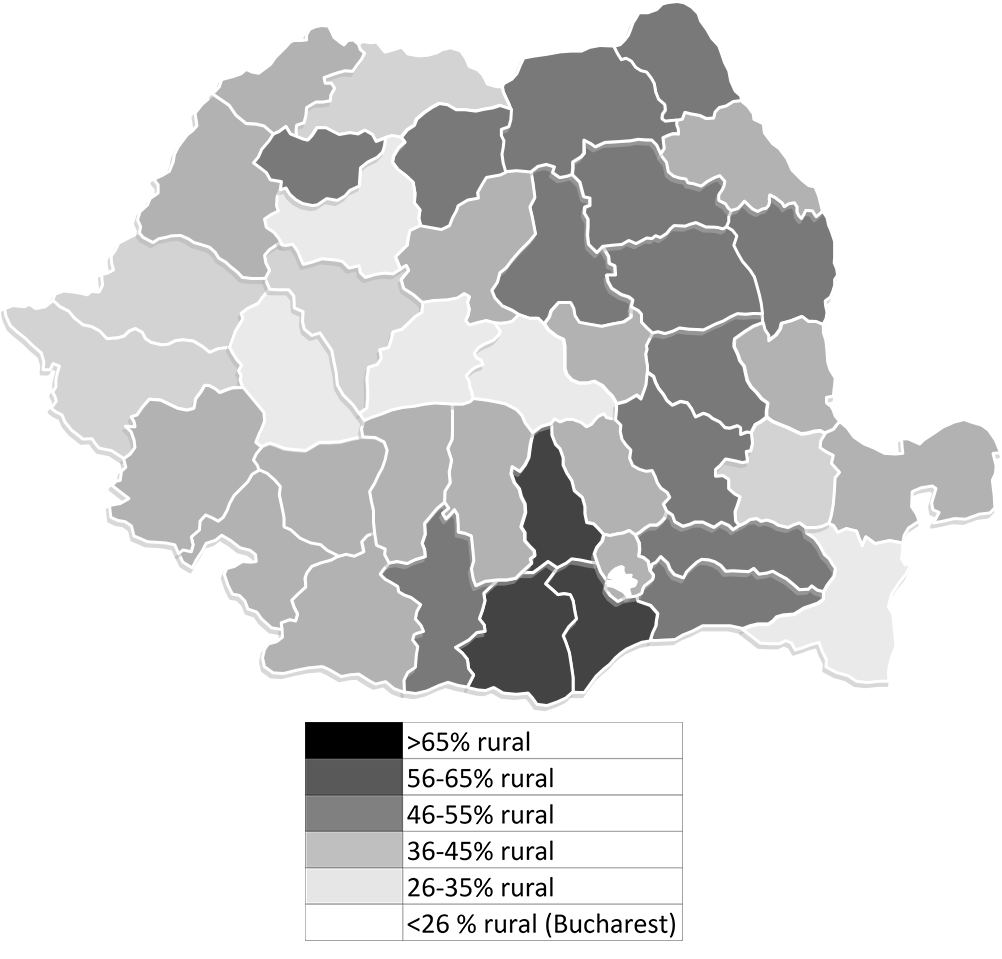


**Romanian healthcare**

Until 1989, Romania had a healthcare system based on the Semashko model introduced in the Soviet Union in 1930 and later adopted by other communist states, characterised by state monopoly, central planning, rigid management, universal coverage and free services. However, because healthcare was considered unproductive (costing money rather than generating revenue) it was chronically underfunded during the Ceaușescu regime, with health spending being even much lower than in other Eastern bloc countries [2]. Healthcare was inefficient and not responsive to patients’ needs [3].

By 1998 the system had been transformed into a social health insurance system, funded by employers and employees [4]. In 2006 national legislation was brought in line with EU law in anticipation of EU membership in 2007 [5]. This health reform law no. 95/2006 is still in place, even though it has been amended many times, mainly through secondary legislation, limiting the effectiveness of health reforms [6]. Health spending in Romania is the lowest in the EU, both per capita (€1,029 as compared to an EU average of €2,884 in 2017) and as a proportion of the GDP (5.2% as compared to an EU average of 9.8% in 2017) [7].

There are also differences in health and healthcare between urban and rural areas. In Cluj County, mortality rate in 2018 was 10.4 in urban areas as compared to 13.9 in rural areas and life expectancy 78.5 as compared to 75.6 [1]. In the whole of Romania, health insurance coverage in 2014 was 94.9% in urban areas as compared to 75.8% in rural areas [8]. In 2018 there was one family doctor per 1,559 urban inhabitants against one per 2,372 rural inhabitants in Cluj County, one hospital bed per 74 urban inhabitants against one per 568 rural inhabitants, one pharmacy per 1,995 urban inhabitants against one per 2,620 rural inhabitants and one dentist per 563 urban inhabitants against one per 2,395 rural inhabitants [1].

**References**

1. Romanian National Institute for Statistics. <http://statistici.insse.ro:8077/tempo-online/#/pages/tables/insse-table>. Accessed 2 April 2019 and 22 March 2021.

2. Bara AC, Heuvel WJA van den, Maarse JAM. Reforms of Health Care System in Romania. Croat Med J. 2002;43(4):446-452.

3. Spiru L, Traşcu RI, Turcu I, Mărzan M. Perpetual transitions in Romanian healthcare. EPMA J. 2011;2(4):341-350.

4. Vladescu C, Scîntee G, Olsavszky V, Allin S, Mladovsky P. Romania: Health system review. Health Systems in Transition 2008;10(3):1-172.

5. Vladescu C, Galan A, Olsavszky V, Scîntee SG. Romanian health system strategic directions for the next decade. Ital J Public Health 2009;7(6).

6. Popescu LG. Analysis of National Health Strategy 2014-2020. Theoretical and Applied Economics Volume XXII 2015;4(605):177-188.

7. OECD/European Observatory on Health Systems and Policies. Romania: Country Health Profile 2019, State of Health in the EU. OECD Publishing, European Observatory on Health Systems and Policies: Paris, Brussels; 2019.

8. Vlãdescu C, Scîntee SG, Olsavszky V, Hernández-Quevedo C, Sagan A. Romania: Health system review. Health Systems in Transition 2016;18(4):1–170.
