# Supplementary material for: Implementation of paediatric vision screening in urban and rural areas in Cluj County, Romania
Source: Int J Equity Health. 2021 Dec 18;20:256. doi: 10.1186/s12939-021-01564-6 (PMC8684067; doi:10.1186/s12939-021-01564-6)
Supplement: Supplementary file 3 — Additional file 3. Questionnaire for screeners. The questionnaire that was distributed among screeners. [file 12939_2021_1564_MOESM3_ESM.docx]

**Additional file 3: questionnaire for screeners**

| **Data** | **Numele cernelii** | |  |  |  |  |  |
| --- | --- | --- | --- | --- | --- | --- | --- |
|  |  |  | |  |  |  |  |
| **INFORMAȚII GENERALE SCREENER** |  |  | |  |  |  |  |
| Muncă experienţă | * <5 ani | * 5 - <10ani | | * 10 - <15ani | * 15 - <20 deani | * > 20 deani |  |
| Volumul de lucru pe săptămână | ... ore |  | |  |  |  |  |
| Lucrez la | * GP | *Gradinita | | * Spital | * Alte | ... |  |
| Eu lucrez ca | * Asistent medical | * Doctor | | * Alte |  |  |  |
|  |  |  | |  |  |  |  |
|  |  |  | |  |  |  |  |
| Estimarea nivelului de conformitate în practică | ... % |  | |  |  |  |  |
|  | **(aproape intotdeauna** | **destul de des** | | **uneori** | **din cand in cand** | **(aproape niciodată** | **nu stiu** |
| Pacientii dor de o programare fără o notificare fai r ly de multe ori | * | * | | * | * | * |  |
| Materialul educațional poate îmbunătăți conformitatea | * | * | | * | * | * | * |
|  |  |  | |  |  |  |  |
|  |  |  |  |  |  |  |  |

| **ATITUDINE PENTRU SCREENINGUL VIZIUNII** |  |  |  |  |  |
| --- | --- | --- | --- | --- | --- |
| Verificarea viziunii trebuie să fie asigurată pentru **toată lumea** din România | * | * | * | * | * |
| Verificarea viziunii trebuie să fie gratuită în România | * | * | * | * | * |
| Cred ca oamenii din Romania doresc ca copiii lor sa fie vizionati | * | * | * | * | * |
| Cred că este important să aflăm dacă pacienții au făcut recomandările pe care le-am prescris | * | * | * | * | * |
| Nu mă îngrijorează de respectarea recomandărilor pacientului meu | * | * | * | * | * |
| Pacienții mei îmi iau întotdeauna recomandările mele de recomandare | * | * | * | * | * |
| Datorită explicației mele, sunt sigur că pacienții mei se conformează recomandării așa cum este prescris | * | * | * | * | * |
| Nu este responsabilitatea mea dacă pacienții nu merg la sesizarea așa cum este prescris | * | * | * | * | * |
| Mi se pare că nu este o problemă dacă pacienții sau părinții lor nu vin la doctori pentru verificarea medicală | * | * | * | * | * |
| Vizionarea slabă a vederii apare din când în când | * | * | * | * | * |
| Nu mă aștept să-mi schimb rutina pentru a îmbunătăți screeningul vizual | * | * | * | * | * |
| Am citit imediat literatura recent publicată despre screeningul vizual | * | * | * | * | * |
| Am nevoie de mai mult timp pentru a aplica măsuri de îmbunătățire a viziunii | * | * | * | * | * |
| Mă întreb uneori dacă pacienții merg într-adevăr la recomandări așa cum esteprescris | * | * | * | * | * |
| Se întâmplă uneori că nu pot explica ce vreau să spun părintelui, deoarece nu vede riscul de ambliopie | * | * | * | * | * |
| Colegii mei și cu mine am discutat tema implementării screening-ului viziunii | * | * | * | * | * |
| Cred că examinarea viziunii este un factor important în munca mea | * | * | * | * | * |
| Abordarea mea față de pacienții care nu participă la consultările de screening nu sa schimbat în ultimul semestru | * | * | * | * | * |
| Sunt dispus să mă confrunt cu nerespectarea prin utilizarea unei alte abordăriîn următoarele câteva luni | * | * | * | * | * |
| Vreau să îmi îmbunătățesc abilitățile de screening pentru viziune | * | * | * | * | * |
| Aș dori să aflu mai multe despre îmbunătățirea screening-ului vizual cât mai curând posibil | * | * | * | * | * |
| A trata cu pacienții care nu primesc ochii lor este o problemă pe termen lung | * | * | * | * | * |
| Dacă identific problemele de vedere ale pacientului, folosesc toate mijloacele posibile pentru a rezolva problema | * | * | * | * | * |
| Im îmbunătățesc tehnicile pe care le folosesc pentru a stimula examinarea vizuală | * | * | * | * | * |
| Încerc să ajut pacienții care nu se prezintă la screening-ul lor cu mai mult de o jumătate de an | * | * | * | * | * |
| Mulți dintre pacienții mei nu au tendința să vină la screeningul vizual | * | * | * | * | * |
| Opinia mea privind pacienții care nu apar pentru screening sa schimbat în ultimul semestru | * | * | * | * | * |
| Se confruntă în mod constant cu o slabă conformitate cu examinarea vizuală | * | * | * | * | * |
| În zilele noastre, îmi pun cunoștințele despre examinarea vizuală în practică, cu o conștientizare mai mare decât în ​​trecut | * | * | * | * | * |
| Am discutat în mod regulat cu colegii mei despre problemele de screening a vederii | * | * | * | * | * |
| În mod automat acord mai multă atenție pacienților care par să nu vadă foarte bine | * | * | * | * | * |
| Spun automat mai mult timp pacienților care nu par să vadă foarte bine | * | * | * | * | * |
| Furnizez în mod consecvent informații suplimentare pacientului | * | * | * | * | * |
| Cred că chestiunea examinării viziunii este evidentă în activitatea mea | * | * | * | * | * |
| Este un obicei de-al meu să-mi păstrez cunoștințele despre viziunea descreening până la data de lectură literatură, conferințe vizita, etc | * | * | * | * | * |
|  |  |  |  |  |  |

| **Întrebări suplimentare** |  |  |  |  |  |
| --- | --- | --- | --- | --- | --- |
|  |  |  |  |  |  |
| Responsabilitatea pentruscreening-ul auzului aparține |  |  |  |  |  |
| E asistenta | * | * | * | * | * |
| Este părinte | * | * | * | * | * |
| Spitalul | * | * | * | * | * |
| Guvernul | * | * | * | * | * |
| Nimeni | * | * | * | * | * |
| În caz de neconformitate, am pus presiune |  |  |  |  |  |
| Este părinte | * | * | * | * | * |
| Spitalul | * | * | * | * | * |
| Medicul de la ORL | * | * | * | * | * |
| Nimeni | * | * | * | * | * |
|  |  |  |  |  |  |
| Am participat la cursul prof.Dr. Vladutiu | * Da | * Nu |  |  |  |

| **Atitudinea față de subcultură** | **tare** **de acord** | **niste** **ce** **de acord** | **nici** **de acord** **nicidezacord** | **dezacordoarecum** | **taredezacord** | **nu stiu** |
| --- | --- | --- | --- | --- | --- | --- |
| În ce măsură sunteți de acord cu următoarele afirmații? |  |  |  |  |  |  |
| România ar trebui să implice toți copiii, inclusiv romii, la screening-ul medical | * | * | * | * | * | * |
| Toți copiii au dreptul la o îngrijire medicală bună, ca orice rezident al României | * | * | * | * | * | * |
| Eu examinez fiecare copil, indiferent de originea lui | * | * | * | * | * | * |
|  |  |  |  |  |  |  |

| **ATITUDINE PENTRU PIERDERILE VIZUALE** | **tare** **de acord** | **niste** **ce** **de acord** | **nici** **de acord** **nicidezacord** | **dezacordoarecum** | **taredezacord** |
| --- | --- | --- | --- | --- | --- |
| În practică nu există copii cu un ochi leneș | * | * | * | * | * |
| Nu știu ce înseamnă cuadevărat un leneș | * | * | * | * | * |
| Nu poți face nimic despre a avea un ochi leneș | * | * | * | * | * |
| Eu vorbesc în mod regulat cu colegii mei despreochii leneși | * | * | * | * | * |
| Știu ce fel de probleme pot fi cauzate de un ochi leneș | * | * | * | * | * |
| Este greu să detectezi unochi leneș | * | * | * | * | * |
| Vreau să fac ceva pentru a ajuta copilul cu un ochi leneș | * | * | * | * | * |
| Opinia mea privind pacienții cu un ochi leneșsa schimbat în ultimul semestru | * | * | * | * | * |
| Se poate face puțin pentru a preveni un ochi leneș | * | * | * | * | * |
| Mi-am schimbat viziunea asupra ochilor lenesi din ultimele 6 luni | * | * | * | * | * |
| Părinții pe care i-am explicat despre un ochi leneș sunt deschiși pentru ca noi să-i examinăm copilul | * | * | * | * | * |
|  |  |  |  |  |  |

| **PĂRINŢI** | **tare** **de acord** | **niste** **ce** **de acord** | **nici** **de acord** **nicidezacord** | **dezacordoarecum** | **taredezacord** |
| --- | --- | --- | --- | --- | --- |
| Îi întreb pe părinți dacă știu ceva despre un ochi leneș | * | * | * | * | * |
| Îi ajut pe părinți să înțeleagă de ce este importantă examinarea vizuală | * | * | * | * | * |
| Verific cum se simt părinții față de un ochi leneș | * | * | * | * | * |
| Verific dacă părinții sunt împotriva examinării vizuale | * | * | * | * | * |
| Părinții ar trebui să fie informați cât mai curând posibil despre programul de examinare vizuală | * | * | * | * | * |
| Părinții ar trebui să primească un prospect care să explice programul de examinare vizuală | * | * | * | * | * |
| Parintii inteleg tot ce imi explic despre screening-ul | * | * | * | * | * |
| Părinții sunt, de obicei, îngrijorați atunci când îiprezint copiii visus | * | * | * | * | * |
| Părinții consideră că examinarea vizuală este importantă | * | * | * | * | * |
|  |  |  |  |  |  |
|  |  |  |  |  |  |

| **CARACTERISTICILE INDIVIDUALE ALE SCREENERILOR** | **(** **aproape** **)mereu** | **destul de** **de multe ori** | **uneori** | **din cand in cand** | **(** **aproape) niciodată** |
| --- | --- | --- | --- | --- | --- |
| eu |  |  |  |  |  |
| La locul de muncă, iau decizii pe cont propriu | * | * | * | * | * |
| Știu cum să rezolv problemele | * | * | * | * | * |
| Lucrez fără sprijinul altora | * | * | * | * | * |
| Știu exact ce trebuie să fac | * | * | * | * | * |
| **Nu** cer sfatul înainte de a lua o decizie | * | * | * | * | * |
| **Nu** am o atitudine „așteptăm și să vedem“ | * | * | * | * | * |
| Mi se pare ușor să facă alegeri | * | * | * | * | * |
| Nu **mă** îndoiesc de diagnosticul pe care îl fac | * | * | * | * | * |
|  |  |  |  |  |  |
| II |  |  |  |  |  |
| Sunt precis în munca mea | * | * | * | * | * |
| Îmi gestionez în mod adecvat volumul de muncă | * | * | * | * | * |
| Lucrez cu atenție | * | * | * | * | * |
| Finalizez tot ce încep | * | * | * | * | * |
| Eu respect cu acordurile făcute | * | * | * | * | * |
| Eu stau în intervalul de timp alocat pentru fiecare vizită | * | * | * | * | * |
| Munca mea se desfășoară de multe ori conform planului | * | * | * | * | * |
| Nu necesită **nici un** efort din partea mea pentru a-mi păstra locul de muncă frumos și ordonat | * | * | * | * | * |
| **Nu** sunt neîngrijit la loculde muncă | * | * | * | * | * |
| Dacă îmi vine în minte ceva, trebuie să o fac imediat, altfel o să uit | * | * | * | * | * |
|  |  |  |  |  |  |
| III |  |  |  |  |  |
| Mă simt strâns asociată cu colegii mei de screening | * | * | * | * | * |
| Mă simt implicat cu pacienții (sau cu părinții) | * | * | * | * | * |
| Iau în considerare dorințele pacientului (sau ale părinților) | * | * | * | * | * |
| Pot să simt bine | * | * | * | * | * |
| Ascult pacientul (sau părinții) bine | * | * | * | * | * |
| Îmi ascult colegii cu atenție | * | * | * | * | * |
| Dau pacientului (sau părinților) suficient timp pentru a pune întrebări | * | * | * | * | * |
| Mi se pare dificil să înțeleg metode noi de examinare străine | * | * | * | * | * |
| Dacă există o nouă metodă de examinare, mă pregătesc bine | * | * | * | * | * |
| Dacă folosesc o nouă metodă de examinare, observ că adesea pacientul / părintele nu cooperează bine | * | * | * | * | * |
| Îmi desfășoară munca în grabă | * | * | * | * | * |
| Imi improvizez cu usurinta | * | * | * | * | * |
| Colaborez plăcut cu ceilalți | * | * | * | * | * |
| Îmi place schimbările | * | * | * | * | * |
| Dacă este necesar, voi prelua imediat munca colegului | * | * | * | * | * |
| Uneori, mi se pare că am luat o muncă inutilă | * | * | * | * | * |
| La serviciu, am timp pentru activități suplimentare | * | * | * | * | * |
| Nu sunt ușor de convins de ceva | * | * | * | * | * |
|  |  |  |  |  |  |
| IV |  |  |  |  |  |
| Îmi păstrez nivelul actual de experiență | * | * | * | * | * |
| Participă activ la asociațiaprofesională națională de screening / asistente medicale | * | * | * | * | * |
| Îi încurajez pe alții să implementeze idei inovatoare | * | * | * | * | * |
| Participă sau doresc să particip, în comitete de asociere | * | * | * | * | * |
| Colaborez fericit cu tranzițiile / schimbările | * | * | * | * | * |
| Rețin ideile inovatoare până când sunt sigur că este ceva bun | * | * | * | * | * |
|  |  |  |  |  |  |
| V |  |  |  |  |  |
| Este clar pentru mine cine este supraveghetorulcolegilor mei de screening | * | * | * | * | * |
| GP / medicul mă trateazăca pe o persoană în poziție inferioară | * | * | * | * | * |
| Dacă supraveghetorul meumă întreabă să fac ceva, o voi face imediat | * | * | * | * | * |
| Consider ierarhia în cadrul depar t ment a fi important | * | * | * | * | * |
|  | **tare** **de acord** | **niste** **ce** **de acord** | **nici** **de acord** **nicidezacord** | **dezacordoarecum** | **taredezacord** |
| Dacă instruiesc un pacient (sau un părinte) să facă ceva, trebuie să se supună | * | * | * | * | * |
| Consider că sunt unul dintre asistenții medicului | * | * | * | * | * |
|  |  |  |  |  |  |
| VI |  |  |  |  |  |
| Dacă nu sunt de acord cu colegii, le pot spune într-o manieră calmă și rezonabilă | * | * | * | * | * |
| Îi felicit pe colegii mei când au făcut ceva corect | * | * | * | * | * |
| Dacă colegii au făcut ceva greșit, pot să le spun calm | * | * | * | * | * |
| Îmi pot exprima furia la locul de muncă fără a-mi pierde controlul | * | * | * | * | * |
| Îmi exprim ideile în timpul întâlnirilor | * | * | * | * | * |
| Mă pot apăra în situații dificile | * | * | * | * | * |
| Sunt critic față de pacienți (sau părinți) | * | * | * | * | * |
| Apel imediat pentru ajutorul colegului meu când nu pot rezolva singur ceva | * | * | * | * | * |
| Dacă vreau să fac un comentariu cuiva, o voi face imediat | * | * | * | * | * |
| Refuz să îndeplinească sarcini delegate care le fac eu nu t dorința de a face | * | * | * | * | * |
| Recunosc greșelile mele | * | * | * | * | * |
| Îi pot spune unui coleg dacă el / ea este nerezonabil | * | * | * | * | * |
| Sunt încrezător în deciziile mele | * | * | * | * | * |
| Pot să le spun colegilor că nu sunt de acord cu ei | * | * | * | * | * |
| Permite altora să-și exprime opiniile, chiar și atunci când nu sunt de acord cu ele | * | * | * | * | * |
| Eu ezită când trebuie să întreb medicul / medicul o întrebare despre un pacient | * | * | * | * | * |
|  |  |  |  |  |  |
| VII |  |  |  |  |  |
| Am o imagine de ansamblu bună despre trebuie sălucrez | * | * | * | * | * |
| Devin ușor tensionat când lucrez și formez tot ce este asociat cu el | * | * | * | * | * |
| Îmi iau cu plăcere conducerea | * | * | * | * | * |
| Îl confrunt cu regularitate pe pacienți (sau părinți) despre comportamentul lor | * | * | * | * | * |
| Adesea spun ceea ce cred | * | * | * | * | * |
| Sunt spontan | * | * | * | * | * |
|  |  |  |  |  |  |

Vă mulțumim foarte mult pentru completarea acestui chestionar!

EUSCREEN - Proiectare de viziune de implementare

Jan Kik și Mandy Nordmann
