# Supplementary material for: Implementation of paediatric vision screening in urban and rural areas in Cluj County, Romania
Source: Int J Equity Health. 2021 Dec 18;20:256. doi: 10.1186/s12939-021-01564-6 (PMC8684067; doi:10.1186/s12939-021-01564-6)
Supplement: Supplementary file 4 — Additional file 4. Questionnaire for rural family doctors. The questionnaire that was distributed among rural family doctors. [file 12939_2021_1564_MOESM4_ESM.docx]

**Additional file 4: questionnaire for rural family doctors**

**CHESTIONAR PENTRU MEDICII DE FAMILIE ÎN ZONELE RURALE DIN JUDEȚUL CLUJ**

| Puteți trimite acest formular prin e-mail la adresa euscreen2020@gmail.com sau, alternativ, completați-l online aici:<https://www.survio.com/survey/d/C5L5B4G2M1G6M2L1P> |
| --- |

Satul sau orașul: ...............................................................................................................................

Numele: .........................................................................................................................................

*Cum ați auzit despre studiul nostru despre screening-ul oftamoligc al copiilor din județul Cluj*?

· anunț la întâlnirea medicilor de familie

· e-mail

· apel telefonic

· nu am auzit despre screening înainte de această scrisoare.

· ............................................................................................................................................................

*Cum evaluați examinarea oculară a copiilor de 4 - 5 ani*:

· Foarte importantă

· importantă

· alte examene sunt mai importante

· nu atât de importantă

· ............................................................................................................................................................

*Ce grupă de vârstă are cel mai mult nevoie de screening oftamologic*?

· Sugari

· copii de vârstă 2-3

· copii preșcolari de vârstă 4-5

· copii școlari

Determinați importanța examenelor de screening ale copiilor preșcolari în vârstă de 4-5, prin adăugarea de numere de la 1 la 8:

..... Creștere

..... Vaccinare

….. Cognitive și mentale

..... Dezvoltare motorică

….. Auz

...... Vorbire

...... Dantură

...... Vedere

*Credeți că părinții vor accepta examinarea oftamologică a copiilor lor*? ................................................

*Credeți că părinții vor cumpăra ochelarii atunci când sunt prescri*ș*i de un oftalmolog*? ......................

*Credeți că copiii vor purta ochelarii*? .......................................................................................................

*Cine ar trebui să efectueze, conform opiniei dvs., screening-ul oftamoligic al copiilor preșcolari în vârstă de 4-5 ani din mediul rural?*

· Medicul de familie? (costuri estimative: .....)Asistenta medicului de familie? (costuri estimative: ....)

· Asistente medicale care să viziteze toatea gr*ă*dini*ț*ele din mediul rural pentru a realiza screening- ul oftamologic în fiecare an?

· ...........................................................................................................................................................

*Ce curs de formare are preferința dvs. (pentru medicul de familie sau asistent*ă *medical*ă):

· 1 zi (6 puncte)

· 2 zile (12 puncte)

· în timpul săptămânii

· sâmbătă

· duminică

·

*Cine ar trebui să finanțeze screening-ul copiilor preșcolari la fiecare 4-5 ani:*

· Ministerul Sănătății

· Ministerul Educației

· ...............................................................................................................................................................

*În Austria, copiii sunt admiși la școală dacă părinții prezintă un "pașaport" cu examenele medicale efectuate (inclusiv screening-ul oftamologic). Ar putea fi un astfel de pașaport medical o idee pentru România*? ............................................................................................................................................................

*Evaluați cele mai importante probleme ale muncii dvs.:*

· Prea mulți pacienți: practica dvs. este pentru ............... locuitori

· Prea multă muncă

· Plata scăzută prin asigurare

· Prea mulți pacienți tratați gratuit

· Prea puține teste sunt rambursate, cum ar fi ECG, teste de sânge și altele, ceea ce necesită prea multe trimiteri

· Creșterea costurilor de personal, precum asistentele medicale

· Călătorii la pacienții din zone izolate

· Reforma întârziată a asistenței medicale în România

· Alte probleme: birocratie foarte mare , incoerenta legislativa ,limitari administrative , conditii de munca improprii fara a putea rezolva singuri aceasta problema ..................................................................................................................................

*Puteți să face*ț*i o estimare a procentului de copii romi în practica dvs.?* ...................%

*Sunteți mulțumit în munca dvs.?*

· Da

· de cele mai multe ori

· uneori

· foarte rar

Vă rugăm să adăugați sugestiile pe care le aveți: ..........................................................................................

........................................................................................................................................................................

Vă mulțumim foarte mult pentru completarea acestui chestionar.
