# Supplementary material for: Implementation of paediatric vision screening in urban and rural areas in Cluj County, Romania
Source: Int J Equity Health. 2021 Dec 18;20:256. doi: 10.1186/s12939-021-01564-6 (PMC8684067; doi:10.1186/s12939-021-01564-6)
Supplement: Supplementary file 5 — Additional file 5. Protocol for measurement of visual acuity, screening protocol and database. A description of the protocols used and the database used to record screening data. [file 12939_2021_1564_MOESM5_ESM.docx]

**Additional file 5: protocol for measurement of visual acuity, screening protocol and database**

A protocol was developed based on literature and expert opinion. Based on the European standard for visual acuity measurement ISO 8596:2017 and expert opinion it was decided to use an optotype chart with logarithmic proportion and a logMAR scale, with positioning of the optotypes as in the ETDRS chart. Tumbling E and Lea Symbols were chosen as optotypes. The use of photoscreening was not considered as there is insufficient evidence that photoscreening is cost-effective, because of poor testability, low positive predictive value and high referral rates [1].

Approximately 200 VA charts were donated by Good-Lite (Elgin, IL, USA), designed for use at three metres, with Tumbling E’s on one side and LEA Symbols on the other side. VA measurement at three metres was considered an appropriate distance for young children. VA was measured in accordance with the aforementioned standard: a line on the chart (0.1 log spacing) is passed when a child indicates three out of five symbols correctly.

VA measurement was done by matching symbols. After the text was explained to the child, the screener put on open spectacles with one eye covered to measure VA of the other eye, with the right eye first. Screeners started using the side of the chart with Tumbling E’s and if the child did not understand the test, they turned the chart to measure VA with Lea Symbols.

The threshold was defined as follows: children aged four passed the test when VA in both eyes separately was 0.2 logMAR (decimal: 0.63) or better, with one line (0.1 log) or less difference between the eyes. Children aged five passed the test when VA in both eyes separately was 0.1 logMAR (decimal: 0.8) or better, with one line or less difference.

Children who did not pass the test were to be referred or to be tested again after one month, the latter when, for example, it was suspected they failed the test because they did not pay attention and not because of actually low VA. When they did not pass the second test, the screeners gave the parents a list of ophthalmologists who had declared to be willing to examine children and to instruct the parents to take the children to their family doctor for referral to an ophthalmologist.

Screeners were provided with packages containing the following items:

- an instruction movie explaining how to measure VA
- an animated instruction movie for the children that the screeners could show before the actual screening took place
- informed consent forms for the parents to sign
- an optotype chart to measure VA
- registration forms to record the results of the examination
- two pairs of examination glasses for the children to wear during screening; one with the right glass covered and one with the left glass covered
- reward stickers for the children to receive after the screening
- a list of ophthalmologists
- referral forms to be filled out by the screener that parents needed to take to the family doctor for referral to an ophthalmologist

To monitor all screening results and follow-up activity of the children that were screened, an electronic database was developed. All stages of the process were to be registered as well as the results of the ophthalmological examination, if the child was referred. In addition, information like birth weight, pregnancy duration, Apgar scores and relevant medical family history was registered, if parents could provide that. All data were collected and registered in accordance with EU regulation 2016/679.

The screening nurses filled out paper forms, because not all were familiar with computers and sometimes local internet access was limited. The forms were entered in the database by the DASM or the UMF-Cluj. Once data were entered in the database, these could not be altered anymore. Instead, an additional record had to be created.

All data were processed in accordance with EU regulation 2016/679: each child was assigned an anonymous code and all personally identifying information (including place of residence) was omitted. The screeners kept separate lists, not accessible to anyone else, to be able to match the children to their codes, in case the results of a repeat screening or diagnostic assessment had to be added to the child’s record in the database. These lists could also be used to eventually check why referred children’s reports of ophthalmological examinations had not been returned.

All screening examination data collected in 2018 were exported from the electronic database on November 29, 2019 for analysis. Most data were entered correctly in the database, with the exception of some errors. A few times secretaries who entered data in the database reported having made an error in data entry, that however could not be corrected because the database did not allow altering recorded data. In addition these self-reported mistakes, there were minor errors such as typos and errors that were obvious because of their internal inconsistency, such as records where the child was, according to the database, not referred but a report of an ophthalmological examination was entered nevertheless, or where the first examination was entered as passed but still a second examination was entered.

While it is not possible to say exactly how many errors were made, based on the available information it is estimated less than 1% of database records were affected by errors.

The fact that first paper forms were filled out by the nurses that were subsequently entered in the database by secretaries, increased the possibility of errors being made. Several interviewed nurses said they would prefer to enter the data themselves, preferably by using a smartphone app, but most of the nurses who attended the courses indicated at the time that they would prefer not to access the database themselves.

**Reference**

1. Rostamzad P, Horwood AM, Schalij‐Delfos NE, Boelaert K, Koning HJ de, Simonsz HJ. Plusoptix photoscreener use for paediatric vision screening in Flanders and Iran. Acta Ophthalmol. 2019;98(1): 80-88.
