# Supplementary material for: Implementation of paediatric vision screening in urban and rural areas in Cluj County, Romania
Source: Int J Equity Health. 2021 Dec 18;20:256. doi: 10.1186/s12939-021-01564-6 (PMC8684067; doi:10.1186/s12939-021-01564-6)
Supplement: Supplementary file 6 — Additional file 6. On-site interviews and questionnaires. Extended analysis of the interviews conducted on-site and the questionnaires distributed among screeners and rural family doctors. [file 12939_2021_1564_MOESM6_ESM.docx]

**Additional file 6: on-site interviews and questionnaires**

Altogether, 34 people were interviewed in January 2019, comprising thirteen family doctors, fourteen nurses and seven kindergarten staff. Of the thirteen doctors, none had screened children even though two had signed a contract with the UMF-Cluj to do so. Of the fourteen nurses, ten had screened children.

The urban kindergartens’ nurses indicated that screening was not difficult because they saw the children on a daily bases and had already established trust. Also, the urban kindergartens are attended by many children, so many children could be screened in a short time.

According to the screeners - both urban and rural - most parents consented. Only a few refused, reportedly out of fear screening might hurt their child, because the child had already been diagnosed with an eye condition, or without providing a reason. How many parents refused is unknown, but there were differences between locations. Some nurses mentioned all parents signed and returned the forms without any problems, while others mentioned that some, or even many, refused or that it took a lot of time and trouble to get the parents to sign and return the forms.

The rural family doctors’ nurses said that it was very difficult to get parents to bring children to the doctor’s office for vision screening. Parents often had other priorities, did not understand the importance, did not think there was anything wrong with their children’s eyes or did not care. There were also several nurses and doctors who mentioned they lacked time to screen and do the paperwork involved.

The nurses who screened at the rural kindergartens, the alternative proposed in August 2018, said this worked better, but was not without problems either. In many communes there are not that many eligible children and the number of children attending the rural kindergartens is usually lower than the number enrolled there. According to kindergarten staff, this is because many parents who go abroad to work, take their children with them or leave their children with grandparents who often live in a different commune. Also, many rural children go to kindergartens in cities, because their parents work there and urban kindergartens usually have a day-long programme (85 out of 114) while most rural kindergartens are only open in the morning (37 out of 42). In winter,  there are even fewer children, according to the kindergarten staff, because many stay at home due to road conditions and seasonal illnesses.

Some nurses said they also tried to screen children at kindergartens in nearby villages, but this approach was hampered by a lack of reimbursement of travel expenses, making it hardly worthwhile to put in a lot of effort to screen a few children. Travelling to other locations was also difficult for nurses without a car, since there is little public transport.

Some nurses and doctors seemed unconvinced or not aware of the benefits of vision screening or did not want anything to do with it for reasons that were unclear. Others were very interested in screening. A few doctors mentioned they had already been screening before the study and some doctors who attended the course found the acquired screening knowledge useful in their general practice. Knowledge about screening appears to play an important part in professionals’ willingness to screen.

Several interviewees who work in communities with either a substantial Hungarian or Roma population mentioned specific problems in reaching these groups. With the Hungarian group, the issue was mainly one of language. While the Romanian language is taught in all schools, not all Hungarians speak the language to the same extent. Especially in rural communes with large Hungarian communities, they may not have a need to use the Romanian language extensively. Written information about the vision screening programme was only available in Romanian, meaning that in cases parents had difficulties with reading leaflets and consent forms, these had to be verbally translated for them.

With the Roma, the biggest issue mentioned was a lack of awareness of the benefits of preventive health care. Several nurses and doctors mentioned that it was sometimes even difficult to convince the Roma to have their children vaccinated. This was not the case in all communes, though.

Views among nurses and doctors differed as to whether parents would take referred children to an ophthalmologist and, if necessary, buy patches or glasses. Most said this would depend on parents’ affluence, though awareness of the necessity was also mentioned as a relevant factor. There was little experience with follow-up in rural areas in the first year of screening though, because few children were screened.

The questionnaires for screeners were distributed in person during the on-site observations. Out of twenty-five questionnaires for screeners distributed, eight were completed, by nurses from Cluj-Napoca and Turda. The questionnaire found a positive attitude towards screening, although some respondents did not look forward to changing their routine to accommodate screening (2 respondents). They did indicate they believe screening is important and should be provided to all children, and felt confident and able to participate in the programme.

After twelve months 40 questionnaires were distributed among screeners again and this time 23 were returned. The attitude of the screeners was similar to the one expressed in the previous questionnaire: they still indicated vision screening is important and should be provided to all children (22), and they considered screening an evident part of their work (21).

The questionnaire for rural family doctors was sent to 98 family doctors and was completed by 23 respondents. The questionnaire was initially sent by postal mail, accompanied by a letter explaining its purpose, and at a later stage doctors who had not yet responded were reminded by telephone and email.

The results shed some light on why coverage was much lower in the rural areas, as compared to Cluj-Napoca and the small cities. In theory, the rural family doctors were very positive about vision screening. Most if not all respondents considered screening for children aged four and five to be very important (20 respondents). The majority believed that parents will accept free examinations (21) and, if necessary, parents would purchase glasses (18) and the children would wear these (20). Several respondents voiced doubts, though. One said whether the parents would buy glasses would depend “on the financial possibilities of the family and the parents' understanding of the need to use glasses by children” and that whether the children would wear the glasses would depend “on the education of parents and children”.

All respondents mentioned many different problems affecting their own work. Most often mentioned was the lack of reform of the national health care system (14). Various other, mostly cost-related problems were also mentioned several times: low payments by insurance, too few diagnostic tests reimbursed and personnel costs. Also mentioned were workload, a lack of awareness of the importance of vision screening among medical professionals as well as among the general population and too many administrative tasks. As one doctor put it, “I would like many aspects of health work to be streamlined and to gain time for interaction with the patient rather than the paper or the computer.”

Doctors also appeared to disagree on who should perform vision screening: some said doctors should do it, others answered nurses and several said both.

Respondents of both questionnaires were made aware of the fact the results would be processed anonymously.
