# Supplementary material for: Implementation of paediatric vision screening in urban and rural areas in Cluj County, Romania
Source: Int J Equity Health. 2021 Dec 18;20:256. doi: 10.1186/s12939-021-01564-6 (PMC8684067; doi:10.1186/s12939-021-01564-6)
Supplement: Supplementary file 7 — Additional file 7. Barriers in rural areas. Extended discussion of the barriers encountered in rural areas. [file 12939_2021_1564_MOESM7_ESM.docx]

**Additional file 7: barriers in rural areas**

Vision screening may not be a priority in rural areas. Some rural nurses did not want to screen, because they were unaware or unconvinced of the benefits, reluctant to do the paperwork or too busy. Many health care professionals have emigrated, especially since the country joined the EU [1] and the shortage of doctors and nurses is far worse in rural areas: less than 20% of Romanian doctors work there and 5% of rural communities have no doctor at all [2] - while 46% of the Romanian population lives in rural areas. In general, Romanian family doctors report limited prevention activity, while nurses are highly occupied with administrative work [3]. Vision screening has to compete for limited resources with other forms of preventive care, such as vaccinations, while health services in rural areas are substandard to begin with [4].

In communes where screening did take place, most parents did not bring their children to the doctor’s office for screening and when screening was extended to rural kindergartens, coverage remained low.

Among the rural population, also, understanding of preventive health care is low; most people only go to the doctor when they are ill. A quarter of Cluj’s rural population has a limited level of health literacy [5]. According to nurses and kindergarten staff, parents often have other priorities than having their children screened, because they do not care or do not understand the importance. This may be why very few parents brought their children to the family doctor’s office for vision screening, even when invited repeatedly. Another barrier may have been that for one-third of the rural population, reaching a doctor’s office requires more than 30 minutes of travel one-way [6].

When it became clear that few children were being screened at the family doctors’ offices, in many communes family doctors’ nurses went to the kindergartens in their communes to screen the children there. Five nurses also screened children in kindergartens in nearby communes. This improved coverage, but was not without its drawbacks either. The enrolment rate in Romania in rural kindergartens is 85%, as compared to 97% in urban kindergartens [7] and the number of children attending is generally lower than the number of children enrolled. This appears to be in part because some people who go abroad to work, take their children with them to another country, while these children are still enrolled in kindergarten. Close to 20% of the working age (20-64) population of Romania is living abroad [8]. When parents leave their children in Romania, they usually leave them in the care of  grandparents [9], who may live in a different commune.

What also may play a role is that many rural parents who have their own farms, have the tendency to keep their children at home more often.

In winter, attendance at the rural kindergartens is lower because children stay at home as a result of illness and weather conditions that lead to roads being impassable. Attendance is also influenced by certain benefits parents and children can receive. When the children attend kindergarten every day, low-income parents can receive RON 50,- (about €11,-) worth of food coupons every month through the ‘Every Child in Kindergarten’ programme [10]. According to kindergarten staff, this has the effect that attendance is higher in the beginning of the school year. Also, at Christmas attendance is higher because the children then receive presents.

In some family doctors’ practices where the nurse could or would not screen, having the family doctor screen instead was considered. However, most family doctors were excluded because of their legal status as an ‘individual medical office’ (CMI) as opposed to a ‘private limited company’ (SRL). The reason for this was that in case of a CMI, a minimum amount of tax would have to be paid per month, regardless of how many children a doctor would screen (RON 670,- or about €141,-). Only if a doctor screened 60 or more children in one month, would the amount of tax due be proportionate. This resulted in low recruitment and almost no doctors screened children. Of the rural family doctors in Cluj County, 70% are CMI’s and 30% are SRL’s [11].

**References**

1. Paina L, Ungureanu M, Olsavszky V. Implementing the Code of Practice on International Recruitment in Romania – exploring the current state of implementation and what Romania is doing to retain its domestic health workforce. Hum Resour Health. 2016;14(1):22.

2. Dumitrache L, Nae M, Dumbraveanu D, Simion G, Suditu B. Contrasting Clustering in Health Care Provision in Romania: Spatial and Aspatial Limitations. Procedia Environ Sci. 2016;32:290–299.

3. World Health Organization. Evaluation of the organization and provision of primary care in Romania. A survey-based project. WHO Regional Office for Europe: Copenhagen; 2012.

4. Vladescu C, Galan A, Olsavszky V, Scîntee SG. Romanian health system strategic directions for the next decade. Ital J Public Health. 2009;7(6).

5. Pop OM, Brînzaniuc A, Sirlincan EO, Baba CO, Chereches RM. Assessing health literacy in rural settings: a pilot study in rural areas of Cluj County, Romania. Glob Health Promot. 2013;20(4): 35–43.

6. Predescu M. Quality in and Equality of Access to Healthcare Services. Country Report for Romania. The Institute of Public Health: Bucharest; 2008.

7. European Commission. Education and Training Monitor 2019 Romania. Publications Office of the European Union: Luxembourg; 2019.

8. Alcidi C, Gros D. (2019), EU Mobile Workers: A Challenge to public finances? CEPS: Bucharest; 2019.

9. Gheaus A. Care drain: who should provide for the children left behind? Crit Rev Int Soc Political Philos. 2013;16(1): 1-23.

10. Fiecare Copil în Gradinita. <http://fiecarecopilingradinita.ro>. Accessed 27 January 2020.

11. Casa Naţională de Asigurări de Sănătate. [http://www.cnas.ro](http://www.cnas.ro/). Accessed 10 April 2019.
